# Antioxidant and Enzyme Inhibitory Properties of the Polyphenolic-Rich Extract from an Ancient Apple Variety of Central Italy (Mela Rosa dei Monti Sibillini)

**DOI:** 10.3390/plants9010009

**Published:** 2019-12-19

**Authors:** Joice Guileine Nkuimi Wandjou, Serena Mevi, Gianni Sagratini, Sauro Vittori, Stefano Dall’Acqua, Giovanni Caprioli, Giulio Lupidi, Giacomo Mombelli, Sabrina Arpini, Pietro Allegrini, Francisco Les, Víctor López, Filippo Maggi

**Affiliations:** 1 School of Pharmacy, University of Camerino, Via S. Agostino 1, 62032 Camerino, Italy; joice.nkuimiwandjou@unicam.it (J.G.N.W.); serena.mevi@studenti.unicam.it (S.M.); gianni.sagratini@unicam.it (G.S.); sauro.vittori@unicam.it (S.V.); giovanni.caprioli@unicam.it (G.C.); giulio.lupidi@unicam.it (G.L.); 2 Department of Pharmaceutical and Pharmacological Sciences, University of Padova, Via Marzolo 5, 35131 Padova, Italy; stefano.dallacqua@unipd.it; 3 Research and Development Department, Indena SpA, 20139 Milan, Italy; giacomo.mombelli@indena.com (G.M.); sabrina.arpini@indena.com (S.A.); pietro.allegrini@indena.com (P.A.); 4 Department of Pharmacy, Faculty of Health Sciences, Universidad San Jorge, Autovía A-23 Zaragoza-Huesca, 50830 Villanueva de Gállego, Spain; fles@usj.es (F.L.); ilopez@usj.es (V.L.); 5 Instituto Agroalimentario de Aragón-IA2 (CITA-Universidad de Zaragoza), Calle Miguel Servet 177, 50013 Zaragoza, Spain

**Keywords:** apple polyphenol-rich extract, HPLC, antioxidant, enzyme inhibition, functional foods

## Abstract

This study was undertaken to evaluate the nutraceutical potential of the Mela Rosa dei Monti Sibillini (MR), an ancient apple variety of the Sibillini Mountains, central Italy. The chemical profile of the apple’s polyphenolic-rich extract (MRE) obtained from first- and second-choice samples using the Amberlite^®^ XAD7HP resin was analyzed by High Performance Liquid Chromatography with Diode-Array and Mass spectrometry (HPLC-DAD-MS) and 21 phytochemicals were quali–quantitatively determined. For comparative purposes, the polyphenol-rich extract of Annurca (ANE), a southern Italian variety, was analyzed. The antioxidant capacity of MREs was evaluated by Folin–Ciocalteu, 1, 1-diphenyl-2-picrylhydrazyl (DPPH), and 2, 2′-azino-bis (3-ethylbenzothiazoline-6-sulphonic acid) (ABTS) assays. The inhibitory capacity of MREs for the enzymes α-glucosidase, lipase, monoamine oxidase A, tyrosinase, and acetylcholinesterase was also determined. The MREs showed higher polyphenolic and triterpene profiles than the ANE. Their radical scavenging activity was higher than that of ANE and comparable to the reference trolox. The MRE from the second-choice apples displayed higher contents of the 21 phytochemicals investigated. Either MRE from second-choice or first-choice samples showed enzymatic inhibition with IC_50_ values higher than those of reference inhibitors but worthy of nutraceutical consideration. Taken together, these results show the potential of MRE as a source of bioactive compounds to be used for pharmaceutical, nutraceutical, and cosmeceutical applications has been confirmed.

## 1. Introduction

The promotion of the recovery of overlooked species is one of the means of the preservation of the ecosystem biodiversity. During the last years, the collection, conservation, and evaluation of fruit tree diversity have received attention in Italy, with the aim of ensuring access to a wide genetic variability and to expand the possibility of diversification of fruit offerings [1]. The characterization and valorisation of old apple cultivars are essential means to inhibit the extinction of potentially useful germplasm [2]. The examination of the pomological descriptions of each variety of these apples is also important, since they are unequal [3]. Thus, old apple cultivars are characterized by unusual pomological traits, such as fruit shape and skin colour, nutritional values, and organoleptic traits, and have a low external attractiveness with respect to commercial apples [1,2]. The loss of importance of the germplasm of these old cultivars in the mind of cultivators and consumers is mainly due to the lack of information on the nutraceutical properties of these old apples and the diffusion of commercial cultivars [1,4]. The high diversity of apple species is also linked to the quality attributes, such as taste, aroma, texture, and phytochemical composition [5].

**Figure 1:**
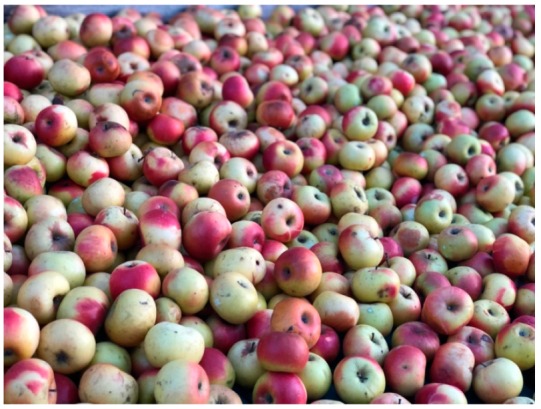
The Mela Rosa dei Monti Sibillini during its traditional storage at ambient temperature.

The Mela Rosa dei Monti Sibillini (MR) (Figure 1) is an ancient apple belonging to *Malus communis* Lam. (Rosaceae), which is cultivated between 400 and 900 m above the sea level (a.s.l.) in the Sibillini Mountains, Marche region, central Italy [6]. This small, flattened apple, endowed with a short peduncle, has been cultivated in this area since the Roman period. Its peel has a greenish colour with shades ranging from pink to purple, an intense and aromatic fragrance, and an acidic and sweet taste. This apple variety is well adapted to the local climate of the Monti Sibillini area [7].

It is widely accepted that healthy diets rich in fruits and vegetables can significantly reduce the risk of cancer and cardiovascular diseases [8]. This may be due to the high abundance of some chemotherapeutic compounds, such as polyphenols and triterpenes, described as secondary bioactive metabolites in these plant foods [3]. Apple is one of the most cultivated fruits in the world, well-known also for its antioxidant properties [3].

During the growth, some young apples are thinned from the tree in order to increase the output and apple quality [9]. Since the organoleptic characteristics are important for the consumers, after harvesting, some apples are discarded due to their undesirable appearance. These discarded apples can lead to a waste-management issue and can be reused due to their high content in polyphenolic compounds [9].

It is suggested that the antioxidant capacities of apples come from the phenolic compounds therein [10]. This activity may be the result of the additive and synergistic effects of all the phytochemicals present in the fruit [10]. The apples polyphenols can be divided into five classes, namely flavan-3-ols/procyanidins, flavonols, dihydrochalcones, anthocyanins, and hydroxycinnnamic acids [11], with quali–quantitative differences depending on the variety and maturation stage [9]. Polyphenols also inhibit carbohydrate hydrolyzing enzymes, such as amylase and α-glucosidase, thus contributing to the decrease of the postprandial hyperglycaemia in the control of diabetes [12,13].

Functional foods with high amounts of bioactive compounds may offer health-promoting advantages and have a role in the prevention of chronic diseases [10]. Separation and purification techniques are needed after crude extraction in order to concentrate the bioactive fraction and discard useless compounds, such as waxes, polysaccharides, and sugars [14]. These extracts are rich in antioxidants and could be used as food supplements to increase the daily intake of health-promoting compounds, and also in the food industry to prolong the food shelf life [15]. Thus, the increasing demand in the food industry for supplements made up of natural antioxidant compounds has stimulated several studies on the purification of extracts from different types of fruits in order to enhance their nutraceutical properties [15,16].

The aim of this work was to evaluate the pharmaceutical, nutraceutical, and cosmeceutical potential of the extracts from the Mela Rosa dei Monti Sibillini (MREs), purified by using a hydrophobic resin, through a comprehensive HPLC analysis followed by measurement of their antioxidant capacity and enzyme inhibitory properties against the α-glucosidase (α-GLU), lipase, monoamine oxidase A (MAO-A), acetylcholinesterase (AChE), and tyrosinase (TYR) enzymes. In order to shed lights on the possible reuse and recovery of discarded apples, MREs obtained from first- and second-choice apples were compared. Finally, the MREs were compared for the phytochemical profile and antioxidant capacity with that of Annurca (ANE), a traditional variety of southern Italy that is currently used in the nutraceutical and cosmeceutical industries.

## 2. Results

### 2.1. Chemical Composition Profile

The whole apple fruit samples of each variety were subjected to warm extraction using ethanol as the solvent. The extract was then purified using the hydrophobic resin XAD7HP to provide polyphenolic-rich extracts (MREs). HPLC-DAD-MS analyses were carried out in order to study possible differences between the first- and second-choice MREs. Moreover, the purified extract of another variety (Annurca, ANE) was used for comparative purposes. The quali- and quantitative analyses were done with 21 compounds belonging to 6 classes, namely flavan-3-ols/procyanidins (catechin, epicatechin, procyanidin A2, procyanidin B2), flavonols (rutin, quercetin, quercetin-3-D-galactoside, kaempferol, kaempferol-3-glucoside), anthocyanins (cyanidin-3-glucoside), phenolic acids (*p*-coumaric acid, neochlorogenic acid, chlorogenic acid, caffeic acid, gallic acid, trans-ferulic acid), dihydrochalcones (phloretin and phloridzin), and triterpenes (annurcoic acid, oleanolic acid, and ursolic acid). The results are reported in the Table 1.

The second-choice MRE was the richest sample, with a total concentration of the studied compounds amounting to 222,892.6 mg/kg (22.3%), followed by the first-choice MRE (203,725.1 mg/kg, 20.4%) and finally the ANE (161,134.4 mg/kg, 16.1%). The most represented polyphenolic compound in the second-choice MRE was epicatechin (42,925.6 mg/kg, 4.3%), followed by chlorogenic acid (34,787.4 mg/kg, 3.5%), procyanidin B2 (30,747.9 mg/kg, 3.1%), and phloridzin (22,066.9 mg/kg, 2.2%), respectively. In the first-choice MRE, epicatechin was also the most represented polyphenolic compound (38,754.3 mg/kg, 3.9%), followed by chlorogenic acid (31,786.1 mg/kg, 3.2%), procyanidin B2 (21,692.8 mg/kg, 2.2%), and catechin (20,914.5 mg/kg, 2.1%). In the ANE, chlorogenic acid was the most abundant phenolic compound (65,753.3 mg/kg, 6.6%), followed by epicatechin (12,303.2 mg/kg, 1.2%) and procyanidin B2 (13,123.0 mg/kg, 1.3%). Some other compounds, such as cyanidin-3-glucoside (798.6 mg/kg), quercetin (582.0 mg/kg), and neochlorogenic acid (637.8 mg/kg), were more concentrated in the ANE than in the first-choice MR (44.9, 18.7, and 325.9 mg/kg, respectively) and the second-choice MRE (85.1, 220.7, and 166.5 mg/kg, respectively).

Three triterpene acids, namely annurcoic, ursolic, and oleanolic acids, represented 4.3–5.2% (52,314.1, 42,975.1, and 42,971.0 mg/kg in second-choice MRE, ANE, and first-choice MRE, respectively) of the quantified compounds, with the most abundant being ursolic acid (20,571.3, 18,864.6, and 18,097.4 mg/kg, respectively), followed by annurcoic acid (23,293.5, 15,382.9, and 17,262.3 mg/kg, respectively) and oleanolic acid (8449.3, 8728.6, and 7611.4 mg/kg, respectively).

### 2.2. In Vitro Antioxidant Capacity Assays

The antioxidant capacities of the three apple polyphenolic-rich extracts (first-choice MRE, second-choice MRE, and ANE) were assessed through Folin–Ciocalteu, DPPH, and ABTS assays. The results are reported in the Table 2.

The analyses of the total polyphenol contents (TPC) showed that the first-choice MRE was the richest (740.0 mgGAE/g), followed by the second-choice MRE (547.1 mgGAE/g) and ANE (517.0 mgGAE/g).

This high TPC of the first-choice MRE was also correlated in the DPPH and ABTS assays, with trolox equivalent antioxidant capacity (TEAC) values equal to 611.4 and 505.8 mgTE/g, and with IC_50_ values of 9.9 and 6.6 µg/mL, respectively. For the second-choice MRE, the values obtained from the DPPH assay were 505.8 mgTE/g (TEAC) and 12.0 µg/mL (IC_50_); in the ABTS assay, they were 643.3 mgTE/g (TEAC) and 7.0 µg/mL (IC_50_), respectively. The ANE displayed lower values, namely 172.6 mgTE/g (TEAC) and 26.4 µg/mL (IC_50_) for DPPH assay, and 402.3 mgTE/g (TEAC) and 12.0 µg/mL (IC_50_) for ABTS assay.

The ABTS assay also demonstrated that the IC_50_ values of the polyphenolic-rich extracts (6.6–12 µg/mL) were very close to that of the reference trolox (4.7 µg/mL), thus showing the impact of the purification on the increase of the antioxidant capacities of the aforementioned extract. Noteworthy, the MREs displayed about a two-fold higher activity than ANE (IC_50_ of 6.6–7.0 µg/mL vs. 12.0 µg/mL, respectively).

### 2.3. Inhibitory Activities on Biological Enzymes

**Figure 2:**
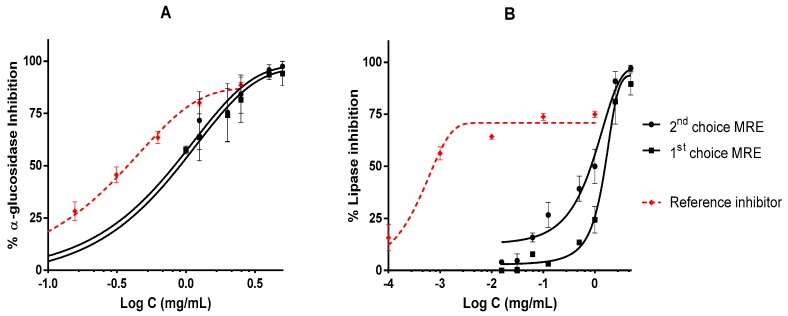
MRE purified extracts inhibit biologically relevant enzymes of metabolic disorders: (**A**) α-glucosidase (α-GLU) inhibition by MREs and acarbose as reference inhibitor; (**B**) lipase inhibition of both MREs and orlistat as reference inhibitor. All experiments are repeated at least in triplicate, and IC_50_ values were calculated by non-linear regression.

The MREs were effective in the inhibition of enzymes involved in the metabolism of carbohydrates and lipids. The extracts were able to inhibit α-GLU in a dose-dependent manner with a similar profile of acarbose, which is the reference inhibitor (Figure 2A). Moreover, there were no significant differences between the IC_50_ values of these samples (first- or second-choice MREs). In the lipase inhibition assay, there were high differences between the reference inhibitor orlistat and the MREs (Figure 2B). However, the MREs had the ability to inhibit the lipase at 100%, while orlistat only reached 75% of inhibition.

**Figure 3:**
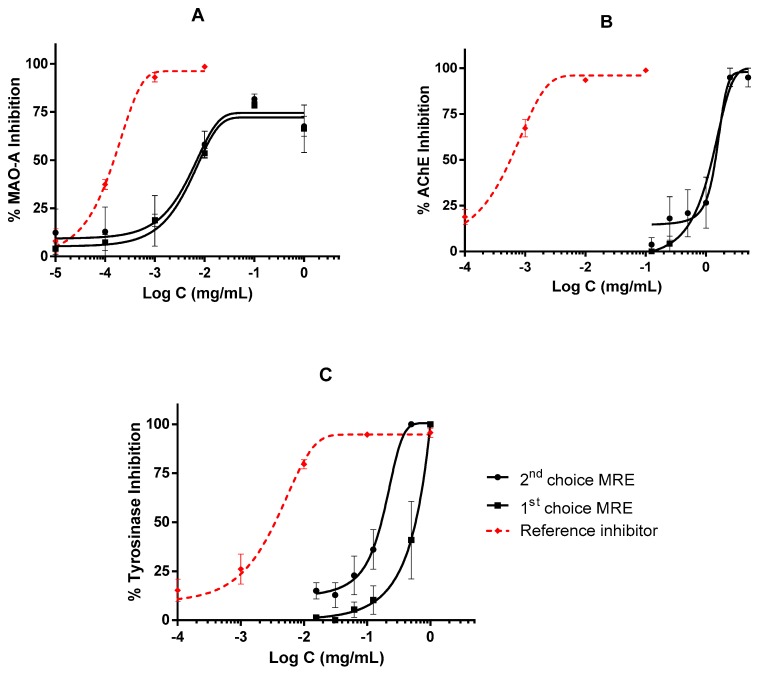
Activity of MREs on enzymes of the CNS: (**A**) MAO-A inhibition by MREs and clorgyline as reference inhibitor; (**B**) AChE inhibition by MREs and galantamine as reference inhibitor; (**C**) TYR inhibition performed by MREs and kojic acid as reference inhibitor. All experiments are repeated at least in triplicate, and IC_50_ values were calculated by non-linear regression.

The extracts were also capable of inhibiting enzymes related to Central nervous system (CNS) in a dose-dependent manner. In Monoamine oxydase A (MAO-A) inhibition assay, the MREs reached 75% of inhibition (Figure 3A), while in AChE and TYR enzymes, extracts achieved 100% inhibition at high doses (Figure 3B,C). Differences in IC_50_ values between the MREs and reference inhibitors were significant in all these enzymes (Table 3).

## 3. Discussion

After the purification of the whole apple extract of three samples belonging to the two cultivars (Mela Rosa and Annurca) with the XAD7HP resin, the polyphenol-rich extracts were investigated for their phytochemical composition through HPLC-DAD, as well as their antioxidant capacity through Folin–Ciocalteu, DPPH, and ABTS assays.

The purification step was revealed to be effective in increasing the concentration of bioactive compounds in MREs by a factor of 24–26 when compared with the ones previously reported in the crude hydroalcoholic extracts of the same apple variety [6]. In this latter study, the most abundant class was that of flavan-3-ols. The high quantity of polyphenols in the purified extracts could be due to the conditions used during the process of purification and the ability of the Amberlite^®^ XAD7HP sorbent resin to adsorb mainly polyphenol compounds, such as flavan-3-ols [14,17]. In addition, the presence of the seeds in the whole apples used to produce the polyphenol-rich extracts should be taken into consideration, since they are richer in polyphenols than peel (3–4 times) and pulp (10–24 times) [15].

In literature, there is a lack of data on the level of many apple polyphenols in purified apple extracts, since studies quantifying a wide spectrum of polyphenols are rather scarce.

Our findings confirmed that the apple polyphenolic-rich extracts have a higher TPC (517.0–740.0 mgGAE/g) when compared with the crude extracts from different cultivars (0.153–0.341 mgGAE/g) [18].

The chemical analysis showed that the second-choice MRE had a higher content of the investigated compounds than the first-choice MRE. On the contrary, the Folin–Ciocalteu assay highlighted a higher TPC for the first-choice MRE (740.0 mgGAE/g) than the second-choice MRE (547.1 mgGAE/g). These differences in the chemical composition and antioxidant capacities showed that the organoleptic aspect of the fruit may have a correlation with its bioactive compounds. In addition, the qualitative and quantitative analyses were not exhaustive because of the impossibility of the identification of all compounds contained in apple and the unavailability of the corresponding standards for a complete identification by HPLC techniques [19].

The higher antioxidant capacity of the first-choice MRE in the Folin–Ciocalteu assay was confirmed in the DPPH and ABTS assays, in which this extract showed TEAC values (611.4 and 682.3 mgTE/g, respectively) higher than that of MRE from second-choice samples (505.8 and 643.3 mgTE/g, respectively). When compared with the Annurca variety, in both chemical composition and antioxidant capacity, the MREs appeared to be better than ANE. In fact, the MREs are 3 times more concentrated in flavan-3-ols than the ANE, with a high content of catechin, epicatechin, and procyanidin B2. This abundance of bioactive compounds in the MREs was also observed for the classes of dihydrochalcones and flavonols, with phloridzin and rutin present as major compounds. Due to the richness of chlorogenic acid, the hydroxycinnamic acids class was more represented in the ANE than in the MREs (around 2 times). Furthermore, the ANE showed higher amounts (2–31 times) of other compounds, such as cyanidin-3-glucoside, quercetin, and neochlorogenic acid, than the MRE. The concentration of the triterpene annurcoic acid, a compound firstly identified in the Annurca apple [20], was surprisingly higher in the MREs than in ANE. Annurcoic acid is a more bioavailable form of ursolic acid, given its chemical resemblance and the presence of an additional hydroxylic group. Ursolic acid is an important inhibitor of the NF-kB-mediated inflammatory response [21].

Apart from nutrients such as carbohydrates and fibres, apple contains phytochemicals that may play a crucial role as bioactive compounds in health [22]. It is well-known that diets rich in fruits, vegetables, grains, nuts, and legumes have protective effects in health, and there is a growing body of evidence showing that dietary polyphenols exert antioxidant properties, acting as therapeutic agents involved in the prevention of disorders in which oxidative stress and inflammation may be involved [23]. Previous studies have also demonstrated that polyphenols from other apple cultivars may exert relevant biological effects, such as antimutagenic, antidiabetic, and antioxidant effects [24,25,26,27]. Polyphenolic compounds have been widely studied, as they are considered to be crucial for the prevention of certain diseases, such as metabolic disorders, in particular diabetes and obesity. The beneficial properties could be in relation to the modulation or inhibition of certain physiologically relevant targets, such as lipase, α-glucosidase, or other receptors by polyphenols or triterpenes. For example, the dihydrochalcone phloridzin is a competitive inhibitor of the sodium glucose co-transporter types 1 and 2, thus exerting an antidiabetic action [28]. It has also been reported that catechin and epicatechin reduce hyperglycemia and hepatic glucose output, while quercetin improves insulin-dependent glucose uptake [29]. Some catechin-like flavan-3-ols have been shown to have inhibitory activity against enzymes such as α-glucosidase and lipase [30]. Apple polyphenols, namely oligomeric procyanidins, have also significantly inhibited the pancreatic lipase activity, thus reducing the increase of plasma triglycerides [31]. The triterpene ursolic acid has also been shown to have a considerable lipase inhibitory activity [5]. It is interesting to note that second-choice MR, which was more concentrated in polyphenols and triterpenes, was also more interesting as an enzyme inhibitor.

The MREs have also demonstrated inhibition of enzymes of the CNS. In this regard, polyphenols contained in apple fruit have exhibited neuroprotective properties and antidepressant activity at low doses, since they are able to cross the blood–brain barrier [32,33]. Quercetin and rutin, besides their antioxidant and anti-inflammatory capacities, inhibit the formation of amyloid-*β* (A*β*) and disaggregate A*β* fibrils in Alzheimer’s disease [32]. It is reported that quercetin as well as some polyphenolic-rich extracts inhibit the enzymes anticholinesterase (AChE) and butyrylcholinesterase (BChE), thus improving cognitive abilities in Alzheimer’s disease, supporting a neuroprotective role of polyphenols [32].

Apple phytochemical composition varies between different cultivars, and some changes are also noticed during maturation and ripening of the fruit [34]. While storage has almost no influence on its phytochemicals profile, processing of the raw material can highly affect it, as seen in our study [34].

Our results demonstrated that the purification of the crude extract highly increased the concentration of almost all the bioactive compounds, as well as the antioxidant and biological properties. The synergy of all the concentrated phytochemicals of these polyphenolic-rich apple extracts may lead to great health benefits [10,35].

## 4. Materials and Methods

### 4.1. Preparation and Purification of Apple Extracts

The MR apples were cultivated in the orchards of Montedinove (N 42°57′44″; E 13°36′15″, 480 m a.s.l.), Marche region, central Italy, and harvested at the end of October 2018. This included the first-choice MR, which has all the organoleptic characteristics required by the consumers, such as the shape, colour, and size; and the second-choice MR, which is normally discarded and not sold because of the unpleasant shape and colour with presence of malformations. The Annurca apples were cultivated in the Campania region, South Italy.

In all cases, apples (1500 g) were cut into small pieces, poured in a blender, covered with 3 L of 90% ethanol, and crushed to a mash consistency. This mash was then transferred into a 20 L round-bottom flask, covered with 15 L of 90% ethanol, and stirred at 65–75 °C for 3–4 h. The hot suspension was filtered through a Büchner funnel, and the filtered solution was vacuum concentrated to a final volume of about 1.5 L. The aqueous concentrate was loaded on a column (I.D. × L = 85 mm × 150 mm) filled with 750 mL of Amberlite^®^ XAD7HP sorbent resin (Sigma-Aldrich, Milan, Italy), previously conditioned in water at a flow rate of 1.5–2 BV/h.

The column was washed with 1.5 L of demineralized water, maintaining the same flow rate. The aqueous eluates were discarded. The column was then eluted with 2.25 L of 90% ethanol containing 0.01% of citric acid, at a flow rate of 1.5–2 BV/h. The eluate was collected when its color changed from light yellow to brown. The hydroalcoholic eluate was concentrated to dryness and dried at 50 °C under vacuum for 24 h to yield about 6 g of purified dry apple extract from “Mela rosa” first-choice and Annurca apples, and 9 g from “Mela rosa” second-choice apples.

### 4.2. HPLC Analysis of Polar Constituents

#### 4.2.1. Reagents and Standards

HPLC-DAD-MS analyses were carried out using a Hewlett-Packard HP-1090 Series II (Palo Alto, CA, USA), equipped with a vacuum degasser, a binary pump, an autosampler, and a model 1046A HP photodiode array detector (DAD) and a Trap SL mass spectrometer detector (Bruker, Billerica, MA, USA) equipped with an electrospray ionization (ESI) source. A Synergi Polar-RP C18 (4.6 mm × 250 mm, 4 µm) analytical column from Phenomenex (Cheshire, UK) was used to accomplish the chromatographic separation. The column was preceded by a security cartridge. For HPLC-DAD (diode array detector) analyses, the mobile phase was a mixture of (A) water with 0.1% formic acid (*v*/*v*) and (B) methanol with 0.1% formic acid, flowing at 1 mL/min in gradient conditions: 0 min, 20% B; 0–15 min, 20% B; 15–45 min, 100% B; 45–55 min, 20% B, 55–60 min, 20% B. The column temperature was set to 30 °C and the injection volume was 10 µL. UV spectra were recorded in the range of 210–400 nm for the 21 compounds, where 272 nm was used for gallic acid, 325 nm for chlorogenic acid, neochlorogenic acid, caffeic acid, *p*-coumaric acid, and trans-ferulic acid; 280 nm for (+)-catechin hydrate, (-)-epicatechin, phloretin, and phlorizin; 230 nm for procyanidin A2 and procyanidin B2; 520 nm for cyanidin-3-glucoside; 265 nm for rutin, quercetin-3-D-galactoside and kaempferol-3-glucoside; 365 nm for kaempferol and quercetin; and 210 nm for ursolic, annurcoic, and oleanolic acids. The identification of annurcoic acid was performed by extracting the ions at *m*/*z* 485.5 [M-H]^−^ from the total ion chromatogram (TIC). After the identification of the peak of annurcoic acid at 43.5 min retention time, quantification was performed by using the calibration curve of ursolic acid at 210 nm. In total, 50 mg of the apple extract was dissolved in 1 mL of MeOH and filtrated before the analysis.

#### 4.2.2. Method Validation

The validation of the method was made by determining linearity, repeatability, recovery, limits of detection (LODs), and limits of quantification (LOQs). The injection of 1–50 mg/L of standard solutions at five different concentrations (i.e., 1, 5, 10, 25, and 50 mg/L) into the HPLC/DAD system was done to construct the calibration curves of the analyzed compounds. Five replicates for each concentration were performed, and the relative standard deviation (RSDs) ranged from 1.6% to 3.6% for run-to-run precision and from 2.9% to 6.8% for day-to-day precision. The correlation coefficient of all the calibration curves was greater than 0.9959. The obtained recoveries for all compounds, evaluated by spiking the samples at two different levels of concentration (5 and 25 mg/L) with a standard mixture, were in the ranges of 83–96% and 89–106%, respectively, with a % RSDs in all cases <9% (*n* = 3). Both the LODs and LOQs of the investigated analytes were in the ranges of 0.02–0.42 mg/L and 0.1–1.2 mg/L, respectively. The specificity of the HPLC-DAD method was demonstrated using the stability of the retention time. Reproducibility of the chromatographic retention time for each compound was examined five times per day over a 5-day period (*n* = 25). The retention times using this method were stable, with a percent RSD value of ≤2.01%.

### 4.3. In vitro Antioxidant Capacity Assays

The in vitro antioxidant activity of the different samples was assessed through three assays by means of standard methods using a SPECTROstar Omega (BMG LABTECH GmbH, Ortenberg, Germany) microplate reader. For the preparation of the stock solution, around 10 ± 1 mg of the different samples were diluted in 1 mL of methanol in an Eppendorf. Then, 100 µL of this stocked solution was diluted with 900 µL of methanol for the analyses. All analyses were conducted at least twice.

#### 4.3.1. Quantification of Total Phenolic Content

The Folin–Ciocalteu assay described by Singleton and Rossi with some modifications was the method used to determine the Total Phenol Content (TPC) of the MREs and ANE [36,37,38]. In total, 300 µL of Folin–Ciocalteu reagent (1 mL Folin-Denis’ reagent in 4 mL H_2_O) was introduced in 4 Eppendorfs, in which 100 µL of the aliquots with different concentrations were placed in advance. Finally, 50 µL of a saturated solution of Na_2_CO_3_ were added and the mixture was centrifugated before the insertion into a 96-well microplate. Each well was evaluated using a microplate reader at an absorbance of 765 nm. The results were expressed as the average of 4 measurements. Gallic acid was the standard used to establish the standard calibration curve. The results were expressed as mg of gallic acid equivalents per g (mgGAE/g) of the polyphenolic-rich extract.

#### 4.3.2. Free Radical Scavenging Activity (DPPH Assay)

The free radical scavenging activity was evaluated using 1, 1-diphenyl-2-picrylhydrazyl (DPPH) on microplate analytical assay, as described by Beghelli et al. and Censi et al. [37,38]. First, 50 µL aliquot of the diluted polyphenolic-rich extract solution and standard at concentrations between 0.016 and 1 mg/mL were introduced into the wells of a microplate, followed by the addition of 200 µL of DPPH in methanol. The absorbance of the wells was measured at 517 nm with a microplate reader against a blank prepared using methanol in addition to the DPPH reagent to get rid of any inherent solvent activity.

#### 4.3.3. Radical Cation Decolorization ABTS Assay

The total radical scavenging activity was measured using 2, 2′-azino-bis (3-ethylbenzothiazoline-6-sulphonic acid) (ABTS) assay [37,38]. The ABTS^•+^ solution was freshly prepared by the oxidation of ABTS (10 mg) by MnO_2_ (0.75 g) in the presence of distilled water (4 mL), followed by 30 min of incubation away from light at room temperature. The working solution was obtained by filtration and dilution with methanol of the previous mixture to give an absorbance of AU 1 at 734 nm. The extract concentrations used were the same as for the DPPH assay, and 200 µL of the ABTS solution was added to the wells. After 15 min of incubation, the absorbance of each well was determined at 734 nm. Trolox (6-hydroxy-2,5,7,8-tetramethylchroman-2-carboxylic acid) was the reference compound used in both DPPH and ABTS assays. The antioxidant capacity of all the samples was expressed in mg trolox equivalent/g extract (mgTE/g). The IC_50_, which is the concentration of the tested material required to cause a 50% decrease in initial DPPH/ABTS concentration, was expressed in mg/mL.

### 4.4. Biological Enzymes Inhibitory Activities

#### 4.4.1. Reagents and Chemicals

The chemical reagents of α-glucosidase from *Saccharomyces cerevisiae*, *p*-nitrophenyl glucopyranoside (pNPG), lipase type II from porcine pancreas, *p*-nitrophenyl butyrate (pNPB), vanillic acid, 4-aminoantipyrine, horseradish peroxidase, tyramine, MAO-A, galantamine, acetylthiocholine iodide (ATCI), 5,5′-dithiobis(2-nitrobenzoic acid) (DTNB), Tris, acetylcholinesterase (AChE), levodopa (L-DOPA), and tyrosinase were acquired through Sigma-Aldrich (Madrid, Spain); clorgyline and α-kojic acid were from Cymit quimica (Barcelona, Spain); MgCl_2_·6H_2_O, HCl, NaCl, and potassium phosphate were from Panreac (Barcelona, Spain).

#### 4.4.2. Bioassays Regarding Metabolic Enzymes

Here, the digestive tract enzymes α-GLU and lipase were chosen due to their importance in the metabolism of carbohydrates and fats; α-GLU catalyzes the hydrolysis of glycosidic bonds by generating simple carbohydrates, while lipase catalyzes the hydrolysis of triacylglycerol to glycerol and free fatty acids. Currently, inhibitors of these enzymes are reference drugs for the treatment of diabetes type II (acarbose) or obesity (orlistat). The α-GLU inhibition assay was based on the previous method [39]. Each well of 96-well plate contained a mix of 100 µL of α-GLU (1 U/mL) and 50 µL of samples at different concentrations or the reference inhibitor (acarbose). After 10 min of preincubation, 50 µL of pNPG (3.0 mM solved in phosphate buffer, pH 6.9) were added to start the reaction. Then, incubation at 37 °C for 20 min was made, and the absorbance was measured at 405 nm using a 96-well microplate reader. Control wells contained solvent instead of samples and blanks buffer instead of the enzyme. Blanks were made in order to eliminate background interference. The results of α-GLU inhibition were expressed as percentage of the control, using Equation (1).

Inhibition (%) = [(Abs_control_ −Abs_sample_)/Abs_control_] × 100 (1)

The lipase inhibition assay was based on a previous protocol [39]. Each well of 96-well plate contained a mix of 40 µL of lipase type II (2.5 mg/mL in Tris-Buffer, pH 7.0) and 40 µL of sample or reference inhibitor (orlistat), which were pre-incubated for 15 min at room temperature. Then, 20 µL of pNPB (10 mM in ethanol) was added to each well and incubated for another 15 min at 37 °C. Blanks and control wells were made. Finally, absorbance was read at 405 nm using a 96-well microplate reader. The inhibitory activity of samples and orlistat was calculated using Equation (1).

#### 4.4.3. Bioassays Regarding CNS Enzymes

The CNS enzymes selected were MAO-A, AChE, and TYR, because they are involved in the regulation of neurotransmitter metabolism. MAO-A and AChE inhibitors are classic drugs used to control different CNS pathologies. TYR inhibition may have neuroprotective effects, but it must also be considered as a cosmetic strategy to avoid skin pigmentation. The inhibitory activity against MAO-A was performed using a previously described technique [40]. Each well of a 96-well microplate contained a mix of 50 µL chromogenic solution (0.8 mM vanillic acid, 417 mM 4-aminoantipyrine, and 4 U/mL horseradish peroxidase in 0.2 M potassium phosphate buffer, pH = 7.6.), 50 µL of samples or reference inhibitor at different concentrations, 100 µL of tyramine (3 mM), and 50 µL of MAO-A (8 U/mL). Blanks and control wells were made. The absorbance was read at 490 nm every 5 min during 30 min. Clorgyline was used as reference inhibitor. The MAO-A inhibitory activity of samples and clorgyline was calculated using Equation (1). The Ellman’s method was used to evaluate the AChE inhibition, as previously described [41]. The assay was performed in 96-well microplates, containing a mixture of 25 µL ATCI (15 mM in Milipore water), 125 µL of DTNB (3 mM in buffer (Tris-HCl-NaCl-MgCl_2_·6H_2_O, pH = 8.0), 50 µL of buffer (50 mM Tris-HCl, pH = 8, 0.1% bovine serum), and 25 µL of different samples or reference inhibitor concentrations. Finally, 25 µL of the AChE (0.22 U/L) was added to start the reaction. Control and blanks were also made. Absorbance was kinetically read 13 times every 13 s at 405 nm. The reference inhibitor used was galantamine. The inhibition results were calculated with Equation (1). The TYR inhibition was assessed in 96-well microplates using a described procedure [42]. Each well contained a mixture including 40 µL of L-DOPA (5 mM in 0.2 M potassium phosphate buffer, pH = 6.8), 10 µL of samples or reference compound, 80 µL of buffer (0.2 M potassium phosphate, pH = 6.8), and 40 µL of tyrosinase (200 U/mL in 0.2 M phosphate buffer, pH = 6.8). Controls and blanks were also performed. Absorbance was read at 475 nm. The reference substance was α-Kojic acid. The inhibition results were also elucidated with Equation (1). In all enzyme inhibitory assays, the statistically significant differences (*p* < 0.05) between samples and reference inhibitors were measured by the one-way ANOVA and Tukey multiple comparison test using the software GraphPad Prism v. 8.0.

## 5. Conclusions

In this study, we have quantified, by using HPLC-DAD-MS, 21 marker analytes from the purified polyphenolic-rich extracts of the Mela Rosa dei Monti Sibillini and Annurca cultivars. Actually, we found out that the former is richer than the latter in the investigated analytes. The MRE from the second-choice apples showed a higher content in bioactive compounds (polyphenols and triterpenes) than the one from the first-choice samples, making them an ideal source for reuse and recovery from nutraceutical and cosmeceutical perspectives. Notably, a significative antioxidant and enzyme inhibitory activity was measured for the MREs. These results give new insights for the future implementation of the production chain of MR in the area of Monti Sibillini, central Italy. This old central Italy apple cultivar seems to be suitable as a functional food and as a source of pharmaceutical, nutraceutical, and cosmeceutical agents to be economically valorised on an industrial level.

## Figures and Tables

**Table 1:** Polyphenolic and triterpene compositions (expressed in mg/kg and %) of the purified apple polyphenolic-rich extracts. **Note**: **MRE** = polyphenolic-rich extract of Mela Rosa dei Monti Sibillini; ANE = polyphenol-rich extract of Annurca; RT = Retention time; λ = wavelength; SD = standard deviation; RSD = relative standard deviation.

			MRE						ANE		
			First-Choice			Second-Choice					
	RT	λ									
Compounds (mg/kg)			Mean	SD	RSD%	Mean	SD	RSD%	Mean	SD	RSD%
*Hydroxybenzoic acids*											
Gallic acid	5.9	272	64.5	1.9	3.0	40.6	1.4	3.4	70.8	7.6	10.7
*Flavan-3-ols*											
Catechin	17.6	280	20,914.5	353.9	1.7	13,397.9	357.8	2.7	4667.7	675.9	14.5
Epicatechin	23.9	280	38,754.3	7686.1	19.8	42,925.6	73.0	0.2	12,303.2	387.3	3.1
Procianidin B2	24.3	230	21,692.8	251.3	1.2	30,747.9	171.8	0.6	13,123.0	207.4	1.6
Procianidin A2	29.9	230	5948.8	59.7	1.0	1456.5	37.0	2.5	1709.9	97.0	5.7
*Anthocyanins*											
Cyanidin 3-glucoside	25.8	520	44.9	2.4	5.4	85.1	4.4	5.1	798.6	8.1	1.0
*Flavonols*											
Rutin	31.47	265	14,172.9	378.1	2.7	17,390.6	215.5	1.2	4596.1	248.7	5.4
Quercetin 3-D-galactoside	32	265	4760.3	26.2	0.6	4601.6	120.3	2.6	4284.2	158.3	3.7
Kampferol-3-glucoside	33.6	265	3518.8	35.2	1.0	2531.4	141.2	5.6	3582.7	85.6	2.4
Quercetin	35.8	365	18.7	0.7	3.9	220.7	3.9	1.8	582.0	35.6	6.1
Kampferol	37.8	365	0.0	0.0	0.0	0.0	0.0	0.0	0.0	0.0	0.0
*Hydrocinnamic acids*											
Neochlorogenic acid	10.58	325	325.9	1.0	0.3	166.5	8.7	5.2	637.8	52.3	8.2
Chlorogenic acid	22.3	325	31,786.1	660.6	2.1	34,787.4	358.2	1.0	65,753.3	1125.5	1.7
Caffeic acid	22.9	325	0.0	0.0	0.0	0.0	0.0	0.0	0.0	0.0	0.0
*p*-Coumaric acid	28.9	325	0.0	0.0	0.0	70.7	9.7	0.0	0.0	0.0	0.0
*trans*-Ferulic acid	30.5	325	0.0	0.0	0.0	0.0	0.0	0.0	0.0	0.0	0.0
*Dihydrochalcones*											
Phloridzin	32.7	280	18,714.3	552.1	3.0	22,066.9	8.2	0.0	6011.9	357.0	5.9
Phloretin	36.3	280	37.4	1.0	2.6	89.1	8.6	9.6	38.1	2.9	7.7
*Total Polyphenols*			160,754.1			170,578.5			118,159.3		
*Triterpenes*											
Annurcoic acid	43.1	210	17,262.3	610.8	3.5	23,293.5	111.1	0.5	15,382.9	1273.2	8.3
Oleanolic acid	45.8	210	7611.4	76.0	1.0	8449.3	574.6	6.8	8727.6	32.6	0.4
Ursolic acid	45.9	210	18,097.4	428.8	2.4	20,571.3	193.0	0.9	18,864.6	804.8	4.3
*Total triterpenes*			42,971.0			52,314.1			42,975.1		
*Total mg/kg*			203,725.1			222,892.6			161,134.4		
*Total %*			20.4			22.3			16.1		

**Table 2:** Antioxidant capacity of the pure dried extract and the purified apple polyphenolic-rich extracts.

	FOLIN	DPPH			ABTS		
		TEAC		IC_50_	TEAC		IC_50_
Samples	mgGAE/g	mgTE/g	mmol TE/g	µg/ML	mgTE/g	mmol TE/g	µg/mL
First-choice MRE	740.0 ± 38.7	611.4	2.4	9.9 ± 0.6	682.3	2.7	6.6 ± 0.3
Second-choice MRE	547.1 ± 45.0	505.8	2.0	12.0 ± 0.5	643.3	2.6	7.0 ± 0.0
ANE	517.0 ± 23.1	172.6	0.7	26.4 ± 0.6	402.3	1.6	12.0 ± 0.9
Positive control Trolox				5.3 ± 1.1			4.7 ± 0.2

**Table 3:** IC_50_ values of the extracts and reference inhibitors calculated by non-linear regression.

Enzymatic Inhibition Assay	IC_50_ (µg/mL)		
	First-Choice MRE	Second-Choice MRE	Reference Inhibitor
α-glucosidase	907.2	823.9 **	378.9 (Acarbose)
Lipase	1584.9 ***	844.7 *	0.8 (Orlistat)
MAO-A	8.03 *	6.98 *	0.15 (Clorgyline)
AChE	1259.0 ****	1430.6 ****	0.6 (Galantamine)
TYR	586.1 **	168.4 ^#^	3.5 (Kojic acid)

Significant differences were detected between samples and reference inhibitors using one-way ANOVA and Tukey multiple comparison test. The α-glucosidase assay: ** *p* < 0.01 (second-choice MRE versus acarbose); lipase assay: *** *p* < 0.001 (first-choice MRE versus orlistat), * *p* < 0.05 (second-choice MRE versus orlistat); MAO-A assay: * *p* < 0.05 (first-choice and second-choice MRE versus clorgyline); AChE assay: **** *p* < 0.0001 (first-choice and second-choice MRE versus galantamine); TYR assay: ** *p* < 0.01 (first-choice MRE versus kojic acid), ^#^ *p* < 0.05 (second-choice MRE versus first-choice MRE). Note: MAO-A = monoamine oxidase A; AChE = acetylcholinesterase; TYR = tyrosinase.
